# Supplementary material for: Increased Incidence and Associated Risk Factors of Aspergillosis in Patients with Bronchiectasis
Source: J Pers Med. 2021 May 17;11(5):422. doi: 10.3390/jpm11050422 (PMC8155934; doi:10.3390/jpm11050422)
Supplement: Supplementary file 1 [file jpm-11-00422-s001.zip › jpm-1180774 R1 Supplementary.pdf]

Supplementary Table S1. Sensitivity analyses using different washout periods. Subdistribution hazard ratios of aspergillosis in the bronchiectasis cohort relative to the matched cohort

Washout period	Subdistribution hazard ratio (95% confidence interval)
1 year	5.03 (3.30–7.66)
2 year	4.94 (3.16–7.71)
3 year	4.94 (3.02–8.08)

Supplementary Table S2. Specific diagnostic codes for aspergillosis in the bronchiectasis cohort and the matched cohort

	Bronchiectasis cohort (n = 51)	Matched cohort (n = 45)
Other pulmonary aspergillosis*	22	17
Aspergillosis, unspecified or other forms of aspergillosis*	25	24
Disseminated aspergillosis or invasive aspergillosis*	9	9

*Some patients had more than one disease diagnosis code.
