# Supplementary figures and images for: Increased Incidence and Associated Risk Factors of Aspergillosis in Patients with Bronchiectasis
Source: J Pers Med. 2021 May 17;11(5):422. doi: 10.3390/jpm11050422 (PMC8155934; doi:10.3390/jpm11050422)

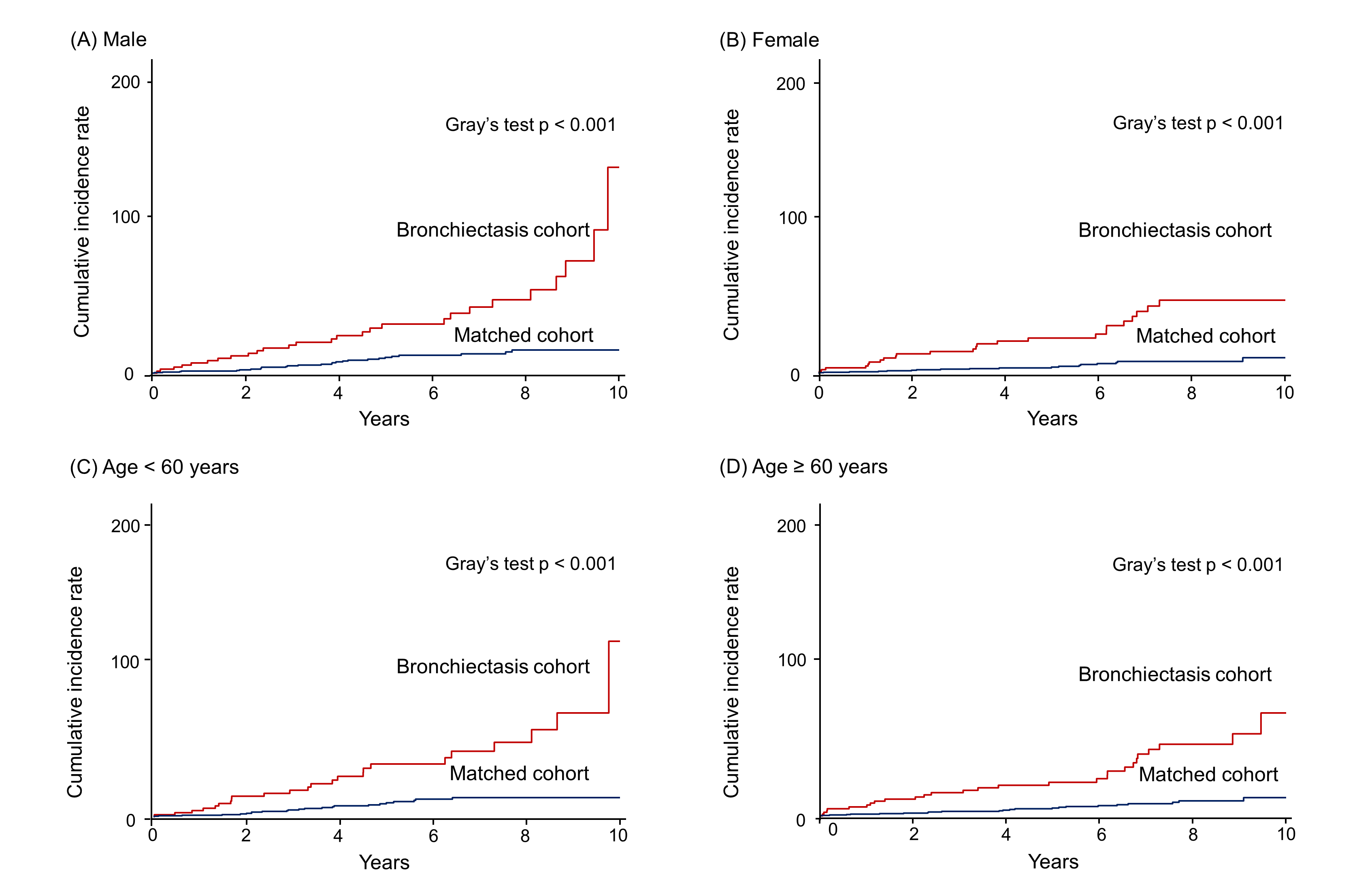

Supplement: Supplementary file 1 [file jpm-11-00422-s001.zip › jpm-1180774 R1 Supplementary Figure S1 20210430.tif]
